# A public backlash towards genomics is a risk all of us working in genomics must share

**DOI:** 10.1016/j.lanepe.2022.100347

**Published:** 2022-03-14

**Authors:** Anna Middleton, Vivienne Parry, Julian Borra, Kate Orviss

**Affiliations:** a Associate Director Engagement and Society, Wellcome Connecting Science, Wellcome Genome Campus, Cambridge, UK; b Director Kavli Centre for Ethics, Science and the Public, Faculty of Education, University of Cambridge, UK; c Head of Engagement, Genomics England, London, UK; d Founder Thin Air Factory, UK; e Senior Advisor (Global Genomics) Public Policy Projects, London, UK

In January 2022, the UK Government Office for Science (GoS) released *Genomics Beyond Healthcare*,^1^ a report with explicit recommendations for ‘greater public dialogue’ around the application of genomic technology.

We see the benefits of genomics through our work in public engagement, communications, ethics and policy. We also see that whole swathes of society actively turn away from genomics due to deep mistrust, lived experience of discrimination, fear of what could happen to their data or being overwhelmed by the science.

Recognition that engagement with public audiences is pivotal to building trust in the technology, and its use among certain communities, is decades old. Yet recently a university project on the genetics of autism caused thousands to sign an online petition as well as stage a physical protest. Protestors saw the word ‘genetics’ but heard ‘eugenics’.^2^

Public audiences do not distinguish between organisations working in genomics. This means a public backlash in one area of genomics impacts all – whether we are in healthcare, in non-profit academic research or for-profit industry developing life-saving gene therapies. Taking proactive steps to understand, explore and address public concerns is something we should be doing collectively because no one organisation can mitigate risk in this arena alone.

Whilst individual organisations often use the science to explain the relevance of genomics (and the GoS report is no exception), we know through our research that the science is very unfamiliar to global public audiences^3^ and the words themselves (‘DNA’, ‘genomics’, ‘sequencing’, ‘variant’) create disconnection rather than connection. Counterintuitively, continually leading with positive news stories about genomics, leads (for some) to cynicism and mistrust.^4^

Meeting the needs of society with respect to genomics requires us to re-frame our engagement from a ‘communications exercise’ to one whose foundational principles are ethics, social science and policy:

1 Ethics – because we need to enable equity of access to genomics for all, which is not possible if even the word itself makes some people turn away;
2 Social science – because we genuinely do not have the evidence base yet to know how to communicate with disconnected audiences at scale as the research on *how* to engage has not been done yet;
3 Policy – because we need a joined-up approach, that crosses organisations within genomics, with recommendations for engagement targeted specifically at disconnected audiences, that can be delivered by all of us, via a unified strategy.

We are calling for all elements of the genomics ecosystem to collaborate in setting guidelines for how to best engage with disengaged public audiences at scale; to test, evaluate and monitor different forms of intervention, to create the evidence base we need to inform future decision-making and to share the operational risk that might arise from a mis-step in another part of the ecosystem.

Our genome is the source code of our humanity and this means that everyone should be empowered to engage with genomics if they want to. Now is the time for us to work together to enable this.

## Contributors

AM, VP, JB and KO contributed equally to the writing of this manuscript. All authors have read and approved the final version of this manuscript.

## Declaration of interests

The authors declare no conflicts of interest.
